# Patterns of mosquito and arbovirus community composition and ecological indexes of arboviral risk in the northeast United States

**DOI:** 10.1371/journal.pntd.0008066

**Published:** 2020-02-24

**Authors:** Joseph R. McMillan, Philip M. Armstrong, Theodore G. Andreadis

**Affiliations:** Environmental Sciences, Center for Vector Biology & Zoonotic Diseases, The Connecticut Agricultural Experiment Station, New Haven, Connecticut, United States of America; Imperial College London, UNITED KINGDOM

## Abstract

**Background:**

In the northeast United States (U.S.), mosquitoes transmit a number of arboviruses, including eastern equine encephalitis, Jamestown Canyon, and West Nile that pose an annual threat to human and animal health. Local transmission of each arbovirus may be driven by the involvement of multiple mosquito species; however, the specificity of these vector-virus associations has not been fully quantified.

**Methodology:**

We used long-term surveillance data consistently collected over 18 years to evaluate mosquito and arbovirus community composition in the State of Connecticut (CT) based on land cover classifications and mosquito species-specific natural histories using community ecology approaches available in the R package VEGAN. We then used binomial-error generalized linear mixed effects models to quantify species-specific trends in arbovirus detections.

**Primary results:**

The composition of mosquito communities throughout CT varied more among sites than among years, with variation in mosquito community composition among sites explained mostly by a forested-to-developed-land-cover gradient. Arboviral communities varied equally among sites and years, and only developed and forested wetland land cover classifications were associated with the composition of arbovirus detections among sites. Overall, the avian host arboviruses, mainly West Nile and eastern equine encephalitis, displayed the most specific associations among mosquito species and sites, while in contrast, the mammalian host arboviruses (including Cache Valley, Jamestown Canyon, and Potosi) associated with a more diverse mix of mosquito species and were widely distributed throughout CT.

**Conclusions:**

We find that avian arboviruses act as vector specialists infecting a few key mosquito species that associate with discrete habitats, while mammalian arboviruses are largely vector generalists infecting a wide diversity of mosquito species and habitats in the region. These distinctions have important implications for the design and implementation of mosquito and arbovirus surveillance programs as well as mosquito control efforts.

## Author summary

The numbers and types of mosquito species in a community vary across space and time, and this variability may be linked to variation in risk of human exposure to mosquito-borne diseases. In this report, the authors use a long-term mosquito-borne virus (i.e., arbovirus) surveillance dataset from the State of Connecticut (CT), United States to first examine whether or not the same land cover classes explain variation in mosquito and arbovirus community composition; they then used statistical models to determine which mosquito species were the most likely and unlikely to test positive for an infection for one of seven arboviruses circulating in CT. Multiple mosquito species tested positive for each arbovirus examined, and blood feeding behaviors were the strongest predictor of whether or not a mosquito species tested positive for a particular arbovirus. Isolations of bird-host arboviruses aggregated in discrete habitat types, while isolations of mammalian-host arboviruses showed no specific habitat associations. The authors conclude that risk of bird-host arbovirus exposures in humans is driven by only a few key mosquito species, whereas risk of mammalian-host arbovirus exposure in humans could be attributed to the general presence and abundance of mammal-feeding mosquito species in the community.

## Introduction

Arboviruses of zoonotic origin continue to cause considerable morbidity and mortality in the United States (U.S.) [1]. West Nile virus (WNV) remains the leading cause of locally acquired arboviral disease, while other mosquito-borne viruses such as Cache Valley virus (CVV), Eastern equine encephalitis virus (EEEV), Jamestown Canyon virus (JCV), and La Crosse virus (LACV) cause sporadic cases with occasional outbreaks [1]. Severe manifestations of human arboviral disease are infrequent; however, clinical diagnosis of several of these arboviruses has steadily increased in the U.S. in recent years [2, 3]. Some arboviruses, such as WNV, have had additional impacts on wildlife health and are increasingly important concerns among conservationists [4, 5]. Additionally, the invasion and range expansion of *Aedes albopictus* (Skuse) as well as the resurgence of *Aedes aegypti* L. in the continental U.S. have increased the potential for introduction and local transmission of dengue, chikungunya, and Zika viruses, all of which currently circulate in tropical U.S. territories [6]. As mosquitoes and their viruses continue to traverse the globe and emerge in unpredictable ways, the U.S. is expected to face an increasing arboviral public health burden in its future [7].

There remain critical knowledge gaps pertaining to the ecology and epidemiology of zoonotic arboviruses that hinder the ability to forecast risk and prevent human exposure. One such knowledge gap is the importance of multi-species infections in the mosquito community. Many of the zoonotic arboviruses circulating in the U.S. are capable of infecting and being transmitted by multiple mosquito species [8, 9]. Epidemiologically, distinguishing when and if arboviral infections in multiple mosquito species represent a risk to humans relies on having an adequate understanding of the mosquito species’ natural histories. The species most likely to perpetuate an arbovirus and drive exposure in humans (i.e., the primary vectors) are those that: 1) blood feed upon appropriate reservoir hosts, 2) are competent for the arbovirus, and 3) are commonly found infected in close spatial and temporal proximity to infections in wildlife hosts [10]. Ecologically, infections in multiple mosquito species could represent variable functional contributions to an arbovirus’ lifecycle: the co-occurrence of multiple mosquito species could amplify epidemics [11] or link epidemics between host populations [12] while, in seasonal climates, competent species that are common during non-epidemic periods could extend the length of transmission seasons [13] and/or increase the probability of the virus surviving inter-epidemic periods [14, 15]. Mathematically, any increase in mosquito species richness could increase the risk of arboviral transmission [16, 17]; however, empirical evidence of such increases are scarce.

Studies that have attempted to link indices of entomological risk to metrics of specific arboviral infections have yielded mixed results. In the WNV system, research in Chicago, Illinois, U.S. did not find a direct relationship between indices of mosquito diversity and WNV infection rates in *Culex* spp. mosquitoes [18], and a study in Atlanta, Georgia, U.S. concluded that WNV infections in non-*Culex* mosquito species likely represent events of vector spillover rather than functional contributions to arboviral transmission [19]. Outside of the WNV system (which is arguably the most-studied zoonotic arbovirus in the U.S.), detecting infections in multiple mosquito species is common–especially among certain mammalian host arboviruses. Longitudinal surveillance of both CVV and JCV in the northeast U.S. has consistently isolated these arboviruses in multiple mosquito species with no clear primary vector species [20, 21]. Research on the mammalian arboviruses is limited in the U.S., and whether or not multi-species infections are an important feature of mammalian arbovirus perpetuation has not been investigated.

If multiple mosquito species are involved in the transmission of an arbovirus, the effort needed to control such a disease increases [22]. Therefore, it is critical to distinguish if and when detected infections represent a realized risk to both humans and wildlife. In this report, we quantified the generality of multi-mosquito species arboviral infections using a long-term mosquito and arbovirus surveillance data set. Mosquitoes and their arboviruses have been monitored in the State of Connecticut since 1996, and over 87 trapping locations have been sampled annually for 18 years. Our study objectives were to: 1) identify drivers of and relationships between variation in mosquito and arbovirus community composition among sites and years, and 2) quantify arbovirus detection likelihoods across mosquito species in order to characterize the general risk of arboviral exposure to human populations in the presence of diverse mosquito communities. Our results provide insights into the ecological and epidemiological complexity of zoonotic arboviruses and inform both the design and implementation of arboviral surveillance and control.

## Methods

### The CAES mosquito and arbovirus surveillance network

**Fig 1:**
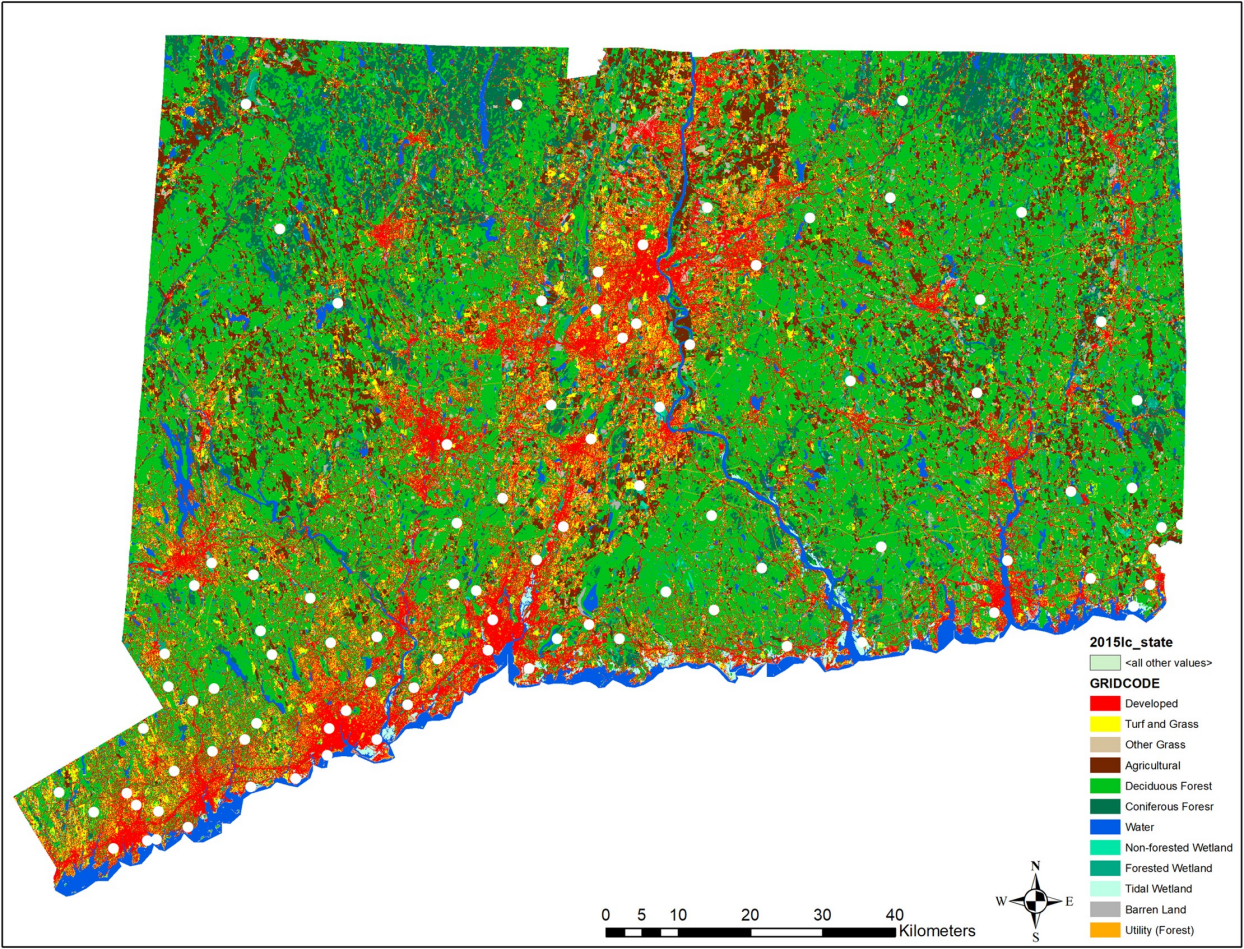
Map of Connecticut 2015 land cover and the Connecticut Agricultural Experiment Station’s mosquito and arbovirus surveillance locations (shown as white circles). Land cover data to create this map were obtained from the University of Connecticut, Center for Land Use Education and Research (CLEAR) (https://clear.uconn.edu/projects/landscape/download.htm#top). The map was created using ArcMap V 10.5.1 (ESRI, Redlands, California, U.S.).

The Connecticut Agricultural Experiment Station (CAES) in New Haven, Connecticut (CT) developed a mosquito and arbovirus surveillance network for detection of multiple arboviruses circulating in CT following an epizootic of EEEV along the Connecticut-Rhode Island border in 1996 [23]. The initial network of trapping sites was mostly located in the eastern half of the state, but with the introduction of WNV in North America and detection of the virus in southwestern CT in 1999 [24], the network was expanded to include the entire state (**Fig 1**). Today, the CAES surveillance network includes 92 locations state-wide which are sampled with CO_2_-baited CDC miniature light traps, gravid traps, and, in certain locations, BG Sentinel Traps on a ten-day trapping schedule from June to October. When an arbovirus is detected, positive sites are sampled twice weekly until there are four consecutive weeks with no arboviral detections. All collected female mosquitoes are morphologically identified to species using a dichotomous key [25], then up to 50 individuals are pooled by collection date and site, trap type, and species. All pools are screened for any arboviral infection using virus isolation techniques in Vero cell culture, and cultures positive for cytopathic effect are tested for a suite of arboviruses, including EEEV, WNV, CVV, Highlands J virus (HJV), JCV, Potosi virus (POTV), Trivittatus virus (TVTV), LACV, and Flanders virus (FLAV) using RT-PCR techniques [26].

### Mosquito and arbovirus community composition metrics

Our first objective was to identify drivers of and relationships between variation in mosquito and arbovirus community composition among sites and years. To better investigate this objective, we first restricted our community ecology analyses to mosquitoes and arboviruses collected using CO_2_-baited light traps only because light traps are considered among the least biased mosquito trapping devices [27]. We also limited our analyses to sites which were sampled in all years of surveillance (n = 87). For analyses of arbovirus community composition, isolations of FLAV and LACV were excluded due to the limited number of isolations of each virus (< 5 total isolates per arbovirus).

Our mosquito and arbovirus community ecology methods relied on numerous functions available in the R package VEGAN [28], and all functions listed in this report were implemented using default settings unless otherwise stated. To examine spatial and temporal patterns of community composition, we analyzed mosquito and arbovirus communities at two different levels: 1) all data aggregated to the site-level, and 2) all data aggregated to the year-level. We first estimated species richness and the Shannon-Wiener index (H, an estimate of species evenness) using the ‘*specnumber’* and ‘*diversity’* functions, respectively. Then, in order to assess how representative mosquito collections were at each site and during each year, we estimated the number of undetected species using the ‘*estimateR’* function, which is an abundance based estimator of species richness [29]; we report biased-corrected Chao estimates. To identify the sites and years contributing most to regional species richness we used the *‘contribdiv’* function. This function returns three components of additive diversity: alpha values which are an estimate of the k^th^ unit’s contribution to within-unit diversity, beta values which are an estimate of the distinctiveness of the k^th^ unit, and gamma values which are an estimate of the k^th^ unit’s total contribution to regional diversity [30]; we report the absolute and unscaled estimates of site-specific and year-specific distinctiveness. To assess how dissimilar mosquito species communities were among sites and years, we estimated the ecological distance among sites and years using the function *‘vegdist*’ (method: Bray-Curtis). We chose the Bray-Curtis index because of this index’s ability to detect underlying ecological gradients [28]. Using *‘vegdist’*, values of 0 indicate complete similarity while values of 1 indicate complete dissimilarity.

To investigate the associations between mosquito species community composition and land cover classifications in a specified area around each trap, we first obtained land cover data for twelve classifications from the University of Connecticut, Center of Land Use Education and Research (https://clear.uconn.edu/projects/landscape/download.htm#top). This file contained information on the amount of area in the state classified as agricultural, barren, coniferous, deciduous, developed, grass, grass (other), forested wetland, non-forested wetland, tidal wetlands, open water, and utility. We then used ArcMap (ESRI) to draw a 2.59 km^2^ buffer (0.51 km radius, buffer area equivalent to 1 square mile) around the geo-location of each trap site. We chose this buffer size assuming, on average and across all mosquito species, a female mosquito flight range of 1 km/night and that each light trap collects a representative mix of species within the 2.59 km^2^ area [31]. We then calculated the percent coverage of each land use classification within each 2.59 km^2^ buffer, and used two different functions in VEGAN to estimate associations among sites and mosquito species as they relate to differences in habitats sampled. We first used ‘*bioenv’* and *‘bioenvdist’* to assess which land cover classifications best correlated with the estimated ecological distances from ‘*vegdist*.’ We then used ‘*metaMDS*’ to implement non-metric multidimensional scaling (NMDS), which is a common unconstrained ordination approach used to visualize multidimensional data in 2-D space. To minimize stress values, we ran ‘*metaMDS*’ with k = 3 dimensions. Data on land cover classifications were then fit to our *metaMDS* results using the ‘*envfit’* function, and results were displayed using the ‘*ordipointlabel*’ function.

We utilized the same functions listed above for our analyses of arbovirus community composition among sites and years. A key difference in these analyses is that arbovirus community composition was analyzed using the number of pools positive for each arbovirus, irrespective of mosquito species identification. Additionally, the comparative rarity of detecting multiple arboviruses versus multiple mosquito species limited our use of certain functions in the VEGAN package. Accordingly, we did not use the *‘estimateR’* function to measure the number of expected viruses at a site because the function uses rare and single species detections in its calculations; we also did not assess site-specific distinctiveness for arbovirus communities. When functions were repeated for arbovirus communities, we compared results with mosquito communities using linear correlations (i.e., Pearson’s r).

### Assessing vector-virus associations

Because mosquito species could be considered “sites” which arboviruses occupy, we calculated the ecological distances between mosquito species based on the number and types of arbovirus isolates identified from gravid and light trap data; species with zero positive isolates of any arbovirus were excluded from this analysis. We then used the ‘*adonis2*’ function to assess which natural history parameters best explained the variance in the estimated distances [32]. Natural history parameters included blood feeding behaviors (mammalian, avian, generalist, other), overwintering strategies (egg, larva, adult), and number of generations in a season (uni- and multi-voltine). Associations between mosquito species based on arbovirus isolations and natural history parameters were then visualized using ‘*metaMDS*’ (k = 2 dimensions), ‘*envfit’*, and ‘*ordipointlabel*’ as described above for mosquito communities.

To better assess vector, site, and epi-week associations for each individual arbovirus, we implemented binomial-error generalized linear mixed effects models (GLMMs) using the ‘*glmer’* function in the R package LMER [33]. In these GLMMs we included data from gravid traps in addition to light traps to account for differences in arbovirus detections by trap type. For each arbovirus, analyses were conducted at the level of the individual pool with the response variable coded as 1 = arbovirus positive and 0 = arbovirus negative. Each GLMM included an intercept offset for pool size, a categorical fixed effect term for trap type (gravid as the reference term), and random intercept effect terms for species, CDC week of collection, year of collection, and collection site. We chose to model species, site, week, and year of collection as random effects because pools collected from the same site, week, year, and/or species are likely to be related in some way due to the nature of spatiotemporally repeated measures and/or intrinsic differences between sites, years, and species. Modeling these terms as random effects also allowed us to assess variability in arbovirus detection among terms rather than in the context of a fixed reference for each variable. Because the beginning and ending dates of seasonal surveillance varied among years, we limited our analyses to data collected between epi-weeks 24 and 41 of each season. Odds ratio estimates for each random effect were generated using the “*get_model_data*” function available in the R package SJPLOT [34].

Finally, we tested for global spatial autocorrelation of arbovirus isolates among sites using the Global Moran’s I spatial auto-correlation function available in ArcMap 10.5.1’s Spatial Statistics tool box (ESRI). The test was implemented using the default settings in ArcMap (conceptualization: inverse distance, distance method: Euclidean, row standardization: false, distance threshold: 25.72 km, spatial weights: not included). All other analyses and plotting functions not specifically mentioned were implemented in R V3.5.1.

## Results

### Mosquito and arbovirus surveillance summaries

In 18 years (2001–2018) of surveillance with CDC light and gravid traps, CAES tested 213,101 pools (3,100,263 individual mosquitoes) with 3,640 pools (1.71%) testing positive for an arbovirus. West Nile virus was the most commonly isolated arbovirus (58.7% of all positive samples): WNV was also isolated in all 18 years of surveillance, at the largest number of surveillance sites (n = 84), and in one-half of all tested species (**S1 Table**). Jamestown Canyon virus was the next most commonly isolated arbovirus, accounting for 11.5% of all virus isolations with detections in every season, at 86.2% of sampling sites, and in over half (52.2%) of tested species. The remaining virus isolates were: POTV (10.2% isolates, 44.4% years, 87.4% sites, 45.7% species), EEEV (8.16% isolates, 66.7% years, 39.1% sites, 39.1% species), CVV (5.22% isolates, 61.1% years, 72.4% sites, 37.0% species), HJV (3.76% isolates, 50.0% years, 36.8% sites, 34.8% species), and TVTV (2.61% isolates, 66.7% years, 23.0% sites, 21.7% species) (**S1 Table**).

The five most commonly collected mosquito species were *Coquillettidia perturbans* (Walker) (17.0%), *Aedes canadensis* (Theobald) (12.4%), *Aedes vexans* (Meigen) (9.75%), *Culex pipiens* L. (9.11%), and *Culex salinarius* Coquillett (7.32%) (**S1 Table**). The most abundant species were not the most commonly infected, and the five species which had the most pools test positive for any arbovirus were *Cx*. *pipiens* (41.2% all arbovirus isolates), *Culiseta melanura* (Coquillett) (10.7%), *Culex restuans* Theobald (8.32%), *Ae*. *canadensis* (6.04%), and *Aedes trivittatus* (Coquillett) (5.44%) (**S1 Table**).

### Mosquito community composition

**Fig 2:**
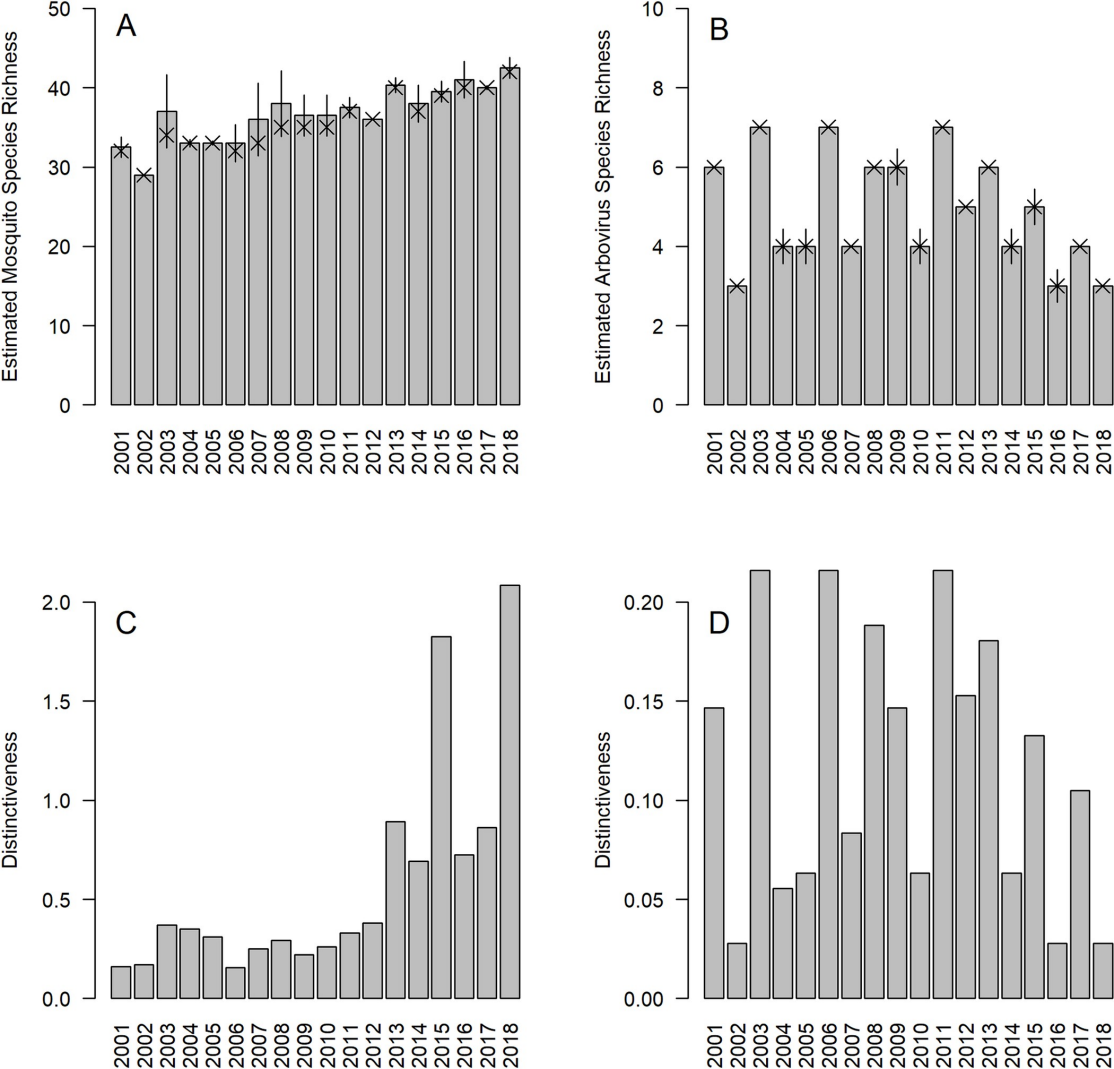
(A and B) Estimated (bars, lines: +/- standard error) and observed (X’s) mosquito species (A) and arbovirus species (B) richness for each year of sampling in Connecticut; (C and D) Year-specific contributions to among unit diversity (i.e., distinctiveness) in mosquito species (C) and arbovirus species (D) richness.

Most mosquito species were widely distributed throughout the state (median number of sites occupied: 78.5, IQR: 27–87), and were captured in every sampling season (median number of years collected: 18, IQR: 10–18). Spatially, the median site-specific mosquito species richness was 30 species (IQR 28–31) with an average evenness estimate of 2.00 (+/- 0.04 SE). Trapping effort was sufficient to detect all likely mosquito species at each site, and observed mosquito species richness was similar to estimated species richness (average difference: 1.55 species (+/- 0.29 SE)). Increasing percentage of coniferous and wetland forest land cover were correlated with increased site-specific distinctiveness (coniferous: r = 0.41, p < 0.001; wetland forest: r = 0.24, p < 0.05); no other land cover classifications nor the variance in land cover composition within a site’s buffer were correlated with site-specific distinctiveness. Temporally, the median annual mosquito species richness was 36 species (IQR 34–39.5) with an average species evenness estimate of 2.60 (+/- 0.03 SE). Similar to site-specific analyses, state-wide mosquito surveillance detected almost all species present in the state each season (average differences between estimated and observed: 0.96 species (+/- 0.25 SE)). Of note was an observed increase in mosquito species richness in CT from 2001 to 2018 (**Fig 2A**). Accompanying this trend was an observed increase in annual ‘distinctiveness’, with the year 2018 being the most distinct season based on mosquito species composition throughout the state (**Fig 2C**).

**Fig 3:**
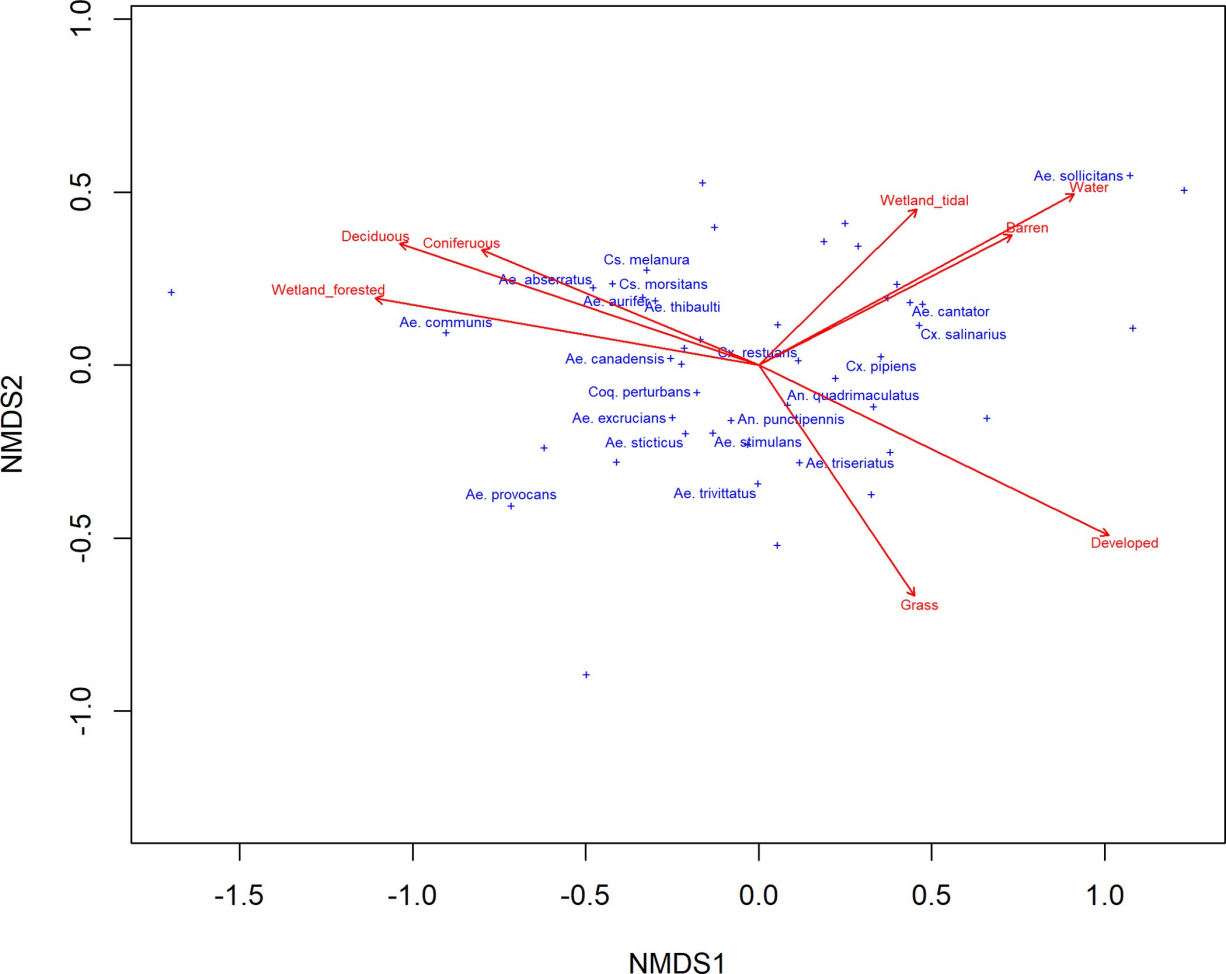
Nonlinear multidimensional scaling plot of the total collection of mosquitoes in light traps at each site across all sampling seasons. Arrows indicate the directional relationship of sites (not shown) and species (blue text and +’s) based on land cover classifications (red text) in the 2.59 km^2^ buffer around each site. Only land class variables significant at p = 0.01 are shown, and only the names of the mosquito species which odds of arbovirus detection’s 95% confidence interval were greater than unity are shown. The label for *Ae*. *taeniorhynchus* (farthest right cross) has been removed for plotting purposes.

Overall, mosquito communities varied more among sites than among years (average ecological distance: sites, 0.67 +/- 0.01 SE; years, 0.34 +/- 0.01 SE). This was not surprising as local scale processes are the primary drivers of mosquito population dynamics [35] and land cover in CT has changed little since 1985 (http://clear.uconn.edu). Variation in the composition of barren, deciduous, developed, grass, open water, forested wetlands, and tidal wetland land cover classifications surrounding each sampling site were correlated with the ecological distances among sites (max correlation, *r* = 0.453). Non-linear multi-dimensional scaling (NMDS) plots, which visually display site by species associations, additionally identified coniferous forests as an important land cover classification associated with differences in mosquito species composition among sites (**Fig 3**, **S2 Table**). The placement of certain mosquito species in **Fig 3** roughly corresponded with known mosquito species natural histories. For instance, *Cx*. *pipiens* associated with more developed habitats, and *Aedes sollicitans* (Walker) and *Aedes taeniorhynchus* (Wiedemann), two brackish water developing species, associated among coastal/water habitats. However, because this analysis utilized adult mosquito collections and habitat associations may be stronger among larval collections, many species showed no obvious associations with any land use classification.

### Arbovirus community composition

In total, at least three arboviruses were detected every sampling season, and at least one arbovirus was detected at each surveillance site (**S1 Table**). Spatially, the median site-specific arbovirus richness was four (IQR: 4–5) with an average evenness estimate of 1.17 (+/- 0.04 SE). Temporally, the median year-specific arbovirus richness was five (IQR: 4–6) with an average evenness estimate of 1.15 (+/- 0.08 SE). Even though greater than 50% of the circulating arboviruses were detected at most sites and during most seasons, evenness estimates indicate that arbovirus communities were either dominated by only one virus or arboviruses were minimally detected. Additionally, unlike with estimates of annual mosquito community richness and distinctiveness, there were no clear temporal trends in arbovirus richness and distinctiveness (**Fig 2B and 2D**).

While mosquito communities were more dissimilar among sites than among years, arboviral communities were similarly different among sites and years (average ecological distance: site 0.63 (+/- 0.01 SE), years 0.58 (+/- 0.02 SE)). The land cover classifications that best correlated with ecological distances in arbovirus communities were different from those correlated with mosquitoes (arbovirus: coniferous, developed, grass, water, and forested/non-forested wetlands) and the magnitude of the correlation was also less for arbovirus communities (maximum correlation, *r* = 0.28). The NMDS plot of site by virus associations further indicated that variability of only a few land cover classifications associated with variation in arbovirus community composition; only developed and forested wetland land cover classifications were associated with site-specific arboviral detections (**S3 Table**). This result may be an artifact of the surveillance network’s inception which was to monitor for WNV and EEEV, which are most common in developed (WNV) and forested wetland (EEEV) habitats, respectively.

Correlations between site-specific metrics of mosquito and arbovirus community composition (i.e., richness, evenness, average ecological distance, and distinctiveness) tended to be positive (**S4 Table**); however, only measures of ecological distance were significantly related (Pearson’s r = 0.36, p < 0.001). This suggests that as sites diverge in mosquito composition they are also likely to diverge in the types of arboviruses detected. Correlations between year-specific metrics of mosquito and arbovirus community composition were more variable and none were significant (**S4 Table**).

### Quantifying vector-virus relationships

In total, forty-seven species of mosquitoes were collected in CT of which thirty-one tested positive for at least one arbovirus (**S1 Table**). The fifteen species which did not test positive for any arbovirus were rarely collected and together accounted for less than 1% of all collected mosquitoes. We found that mosquito blood feeding ecology and number of generations per year were the most important natural history parameters associated with arbovirus isolations in mosquito species (Blood feeding: pseudo-F 2.20, pseudo-p < 0.01; Generations: pseudo-F 3.34, pseudo-p < 0.01). These associations were readily apparent in an NMDS plot: vectors of avian arboviruses were distinct from vectors of mammalian arboviruses, WNV vectors were distinct from EEEV and HJV vectors, univoltine *Aedes* spp. associated with JCV, and finally, there were no clear distinctions in vector species for the remaining mammalian arboviruses (**S1 Fig**).

**Fig 4:**
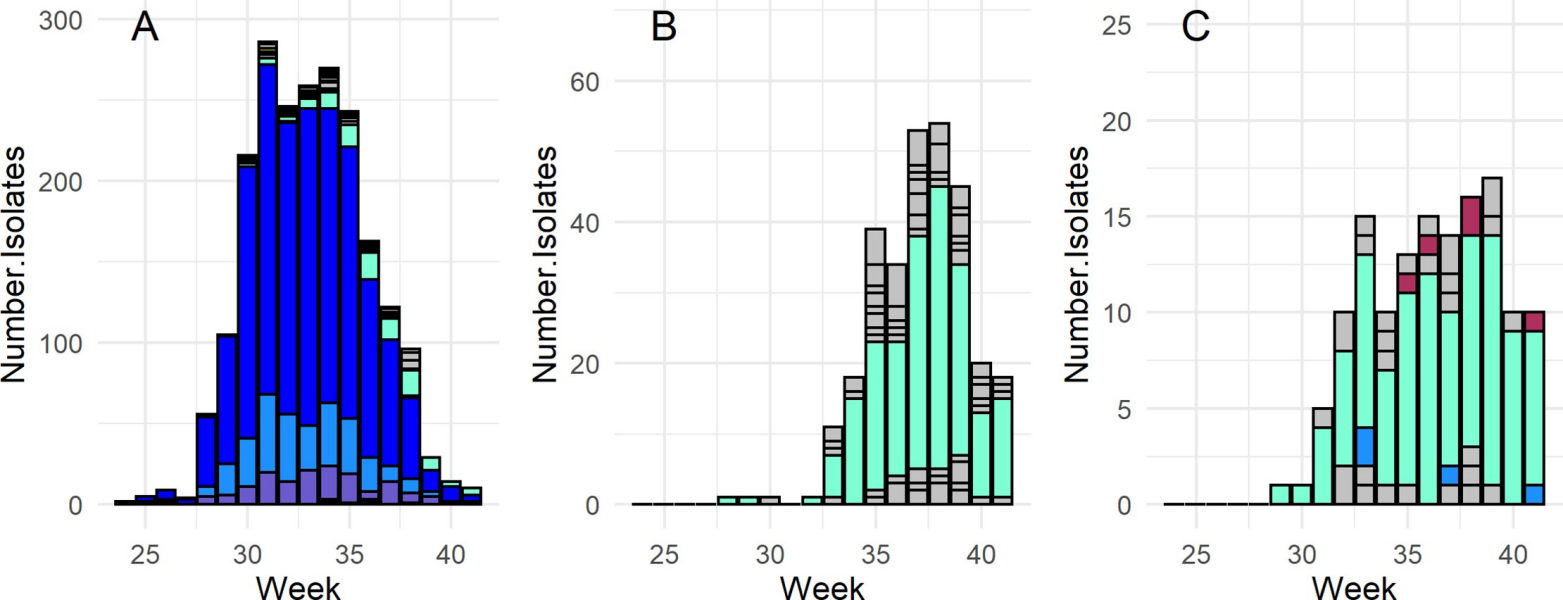
Epidemic curves for the avian arboviruses: West Nile virus (A), eastern equine encephalitis virus (B), and Highlands J virus (C). Only mosquito species with a significant positive association with the arbovirus are shown in color in each plot; all other species are represented in grey.

**Fig 5:**
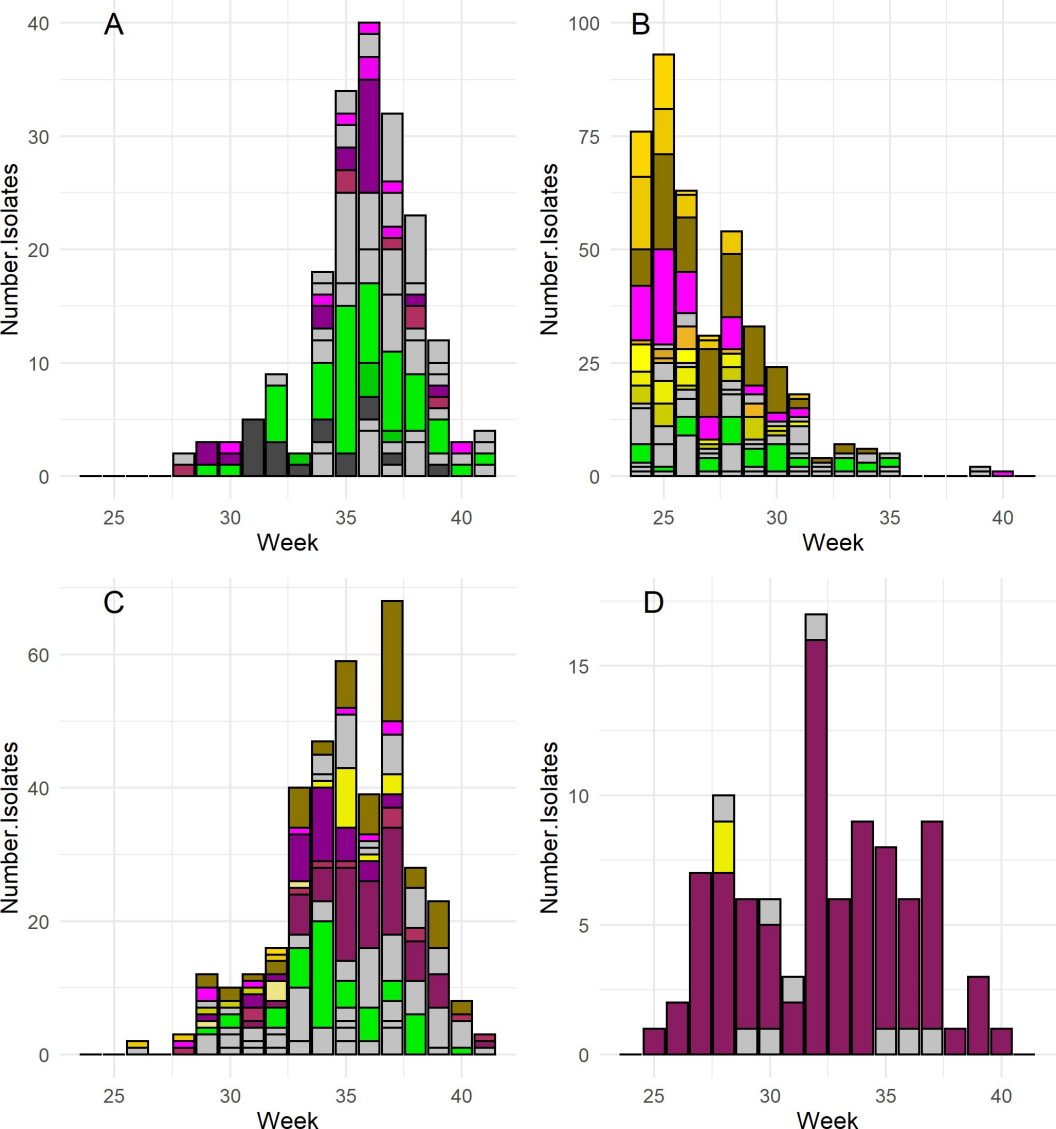
Epidemic curves for the mammalian arboviruses: Cache Valley virus (A), Jamestown Canyon virus (B), Potosi virus (C), Trivittatus virus (D). Only mosquito species with a significant positive association with the arbovirus are shown in color in each plot; all other species are represented in grey.

All arbovirus specific GLMMs further supported the associations among mosquitoes and arboviruses observed in our community ecology analyses (all GLMM tables are available as supporting information). With the exception of EEEV and TVTV, both avian and mammalian arboviruses associated with multiple vector species (**Table 1, Figs 4 & 5**; the legend for these Figures is provided in S2 Fig). It was also clear from our aggregated results that single mosquito species associated with multiple arboviruses (**Table 1**). The existence of primary vector species was evident for EEEV and HJV (*Cs*. *melanura*), TVTV (*Ae*. *trivittatus*), and WNV (*Cx*. *pipiens*) as shown by the total number of isolates, the high odds ratio of samples identified as these species testing positive for each virus, and the prevalence of isolates in each species throughout each virus’ detection period (**Table 1, Figs 4 & 5**). For WNV, our results identified *Cx*. *restuans*, *Cx*. *salinarius*, *Cs*. *melanura*, and *Culiseta morsitans* as likely secondary vectors. Our methods also found a significant odds ratio of WNV detection for the mammal-biting species *Aedes stimulans*, though this association should be interpreted cautiously as it is likely an artifact of the GLMM’s structure and the unusual timing/prevalence of WNV detections in this species at a particular site in a particular year in the data set. We detected no clear primary vectors for CVV, JCV, and PTV as infections in multiple species were dispersed throughout each virus’ detection period and many species across genera associated with these viruses (**Table 1, Fig 5**). Despite the lack of a primary vector, there were clear distinctions in vector-virus associations between JCV and CVV/POTV; univoltine *Aedes* species strongly associated with JCV while multivoltine species across multiple genera associated with CVV and POTV (**Table 1, S1 Fig**).

**Table 1:** Odds that a pool identified to a specific mosquito species tested positive for any of seven arboviruses circulating in Connecticut, U.S. from 2001–2018. Odds ratios were generated from arbovirus-specific binomial error generalized linear mixed effects models with infection status (1 –positive, 0 –negative) as the response variable, pool size as an intercept offset, trap type as a fixed effect term, and site, week, year, and species as random intercept terms. Only odds ratios which 95% confidence limits (CI) did not include unity are shown.

Host Choice	Generations per season	Mosquito Species	Mammalian Arboviruses				Avian Arboviruses		
			Cache Valley	Jamestown Canyon	Potosi	Trivittatus	Eastern Equine	Highlands J	West Nile
Avian	Multi-	*Culex* *pipiens*		0.02 (0.002, 0.14)	0.07 (0.01, 0.36)				30.6 (18.2, 51.2)
		*Culex* *restuans*		0.09 (0.02, 0.44)	0.12 (0.02, 0.61)			1.50 (1.42, 14.2)	26.2 (15.5, 44.2)
		*Culiseta* *melanura*	0.14 (0.03, 0.59)	0.07 (0.006, 0.74)	0.27 (0.003, 0.20)		3.59 (2.37, 5.44)	12.3 (6.18, 24.3)	8.77 (5.02, 15.3)
		*Culiseta* *morsitans*							6.35 (1.29, 31.2)
Generalist	Multi-	*Culex* *salinarius*	0.17 (0.05, 0.58)	0.21 (0.07, 0.66)	0.22 (0.08, 0.65)				2.80 (1.63, 4.83)
	Uni-	*Aedes* *excrucians*		14.6 (6.77, 31.4)					
		*Coquillettidia* *perturbans*	4.20 (1.85, 9.55)	0.44 (0.24, 0.81)					
Mammalian	Uni-	*Aedes* *abserratus*		16.7 (8.75, 31.9)	14.5 (1.10, 191.8)				
		*Aedes* *aurifer*		15.4 (8.50, 27.8)	23.9 (5.28, 108.5)				
		*Aedes* *canadensis*		2.71 (1.64, 4.47)	4.67 (2.01, 10.8)				
		*Aedes* *communis*		25.1 (3.67, 158.2)					
		*Aedes* *provocans*		210.9 (77.8, 571.8)					
		*Aedes* *sticticus*		2.37 (1.19, 4.73)	5.69 (1.12, 15.3)	5.77 (1.19, 28.9)			
		*Aedes* *stimulans*		10.9 (5.72, 20.7)	19.6 (4.61, 83.7)				5.29 (1.12, 24.9)
		*Aedes* *thibaulti*			3.62 (1.08, 12.2)				
	Multi-	*Aedes* *cantator*	3.57 (1.21, 10.6)	14.3 (8.2, 25.0)	6.50 (2.30, 18.4)				
		*Aedes* *sollicitans*	3.65 (1.08, 12.4)						
		*Aedes* *taeniorhynchus*	3.66 (1.51, 8.90)		4.35 (1.70, 11.1)				0.29 (0.14, 0.59)
		*Aedes* *triseriatus*	2.83 (1.01, 7.69)		4.19 (1.59, 11.0)			4.28 (1.44, 12.8)	
		*Aedes* *trivittatus*			5.05 (2.17, 11.8)	100.0 (52.0, 192.4)			
		*Aedes* *vexans*							0.18 (0.07, 0.49)
		*Anopheles* *punctipennis*	20.0 (9.91, 40.5)	11.1 (6.43, 19.1)	9.26 (3.99, 21.5)				
		*Anopheles* *quadrimalatus*	7.37 (2.45, 22.1)						
		*Psorophora* *ferox*							0.29 (0.11, 0.75)
Other (Amphibian/ reptilian)	Multi-	*Uranotaenia* *sapphirina*			0.06 (0.007, 0.59)				

All arboviruses, except TVTV, displayed distinct within-season detection periods (**Figs 4 & 5**). There was also substantial overlap in these detection periods, and the detection periods of CVV, POTV, EEEV and HJV coincided in late summer. Among the mammalian arboviruses, the epidemic period of JCV was distinct from the three other mammalian arboviruses and occurred in early summer, which is likely due to the importance of early spring univoltine species and vertical transmission of the virus [20] (**Fig 5**).

**Fig 6:**
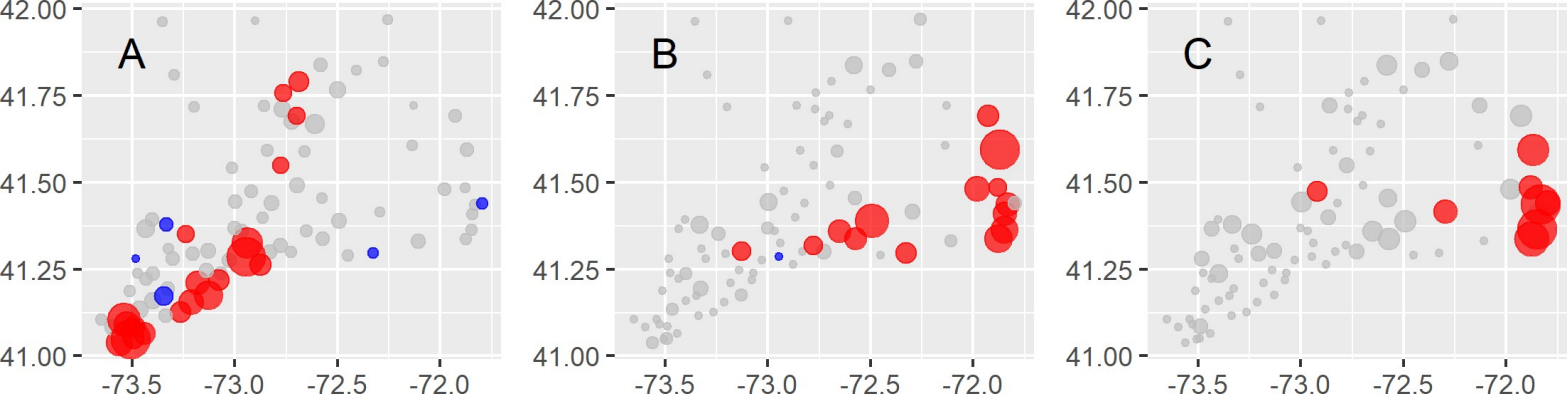
Risk maps for the avian arboviruses: West Nile virus (A), eastern equine encephalitis virus (B), and Highlands J virus (C). Point sizes correspond to the number of isolates scaled to the maximum number of isolates detected while colors indicate whether the 95% confidence interval for the odds of arbovirus detection were > 1 (red), included 1 (grey), or were < 1 (blue).

**Fig 7:**
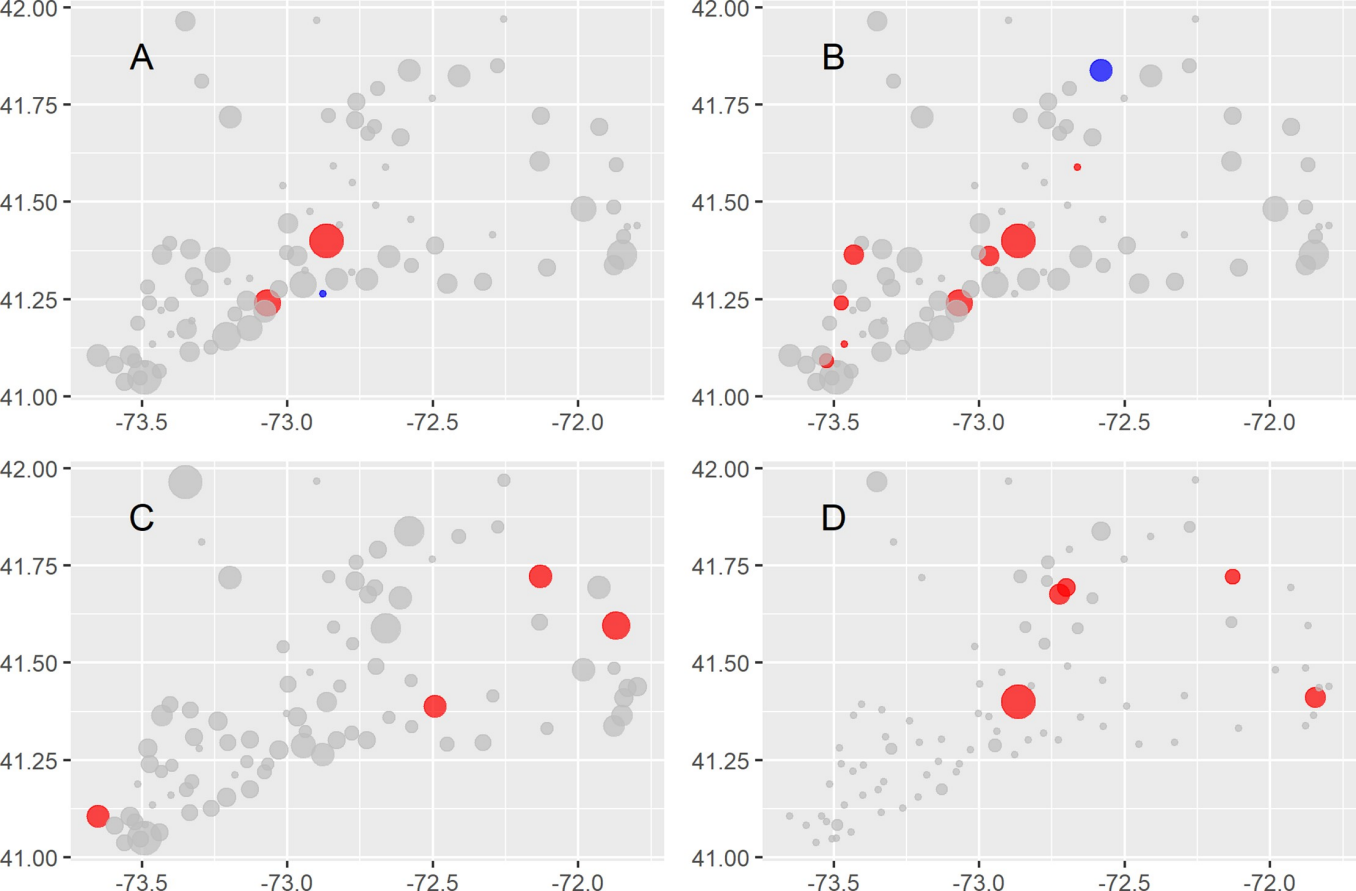
Risk maps for the mammalian arboviruses: Cache Valley virus (A), Jamestown Canyon virus (B), Potosi virus (C), Trivittatus virus (D). Point sizes correspond to the number of isolates scaled to the maximum number of isolates detected while colors indicate whether the 95% confidence interval for the odds of arbovirus detection were > 1 (red), included 1 (grey), or were < 1 (blue).

The avian arboviruses showed the strongest spatial and habitat associations among the seven arboviruses. West Nile virus was more common in the southwestern region of CT (**Fig 6A**) which is also the most developed in the state; these sites are also likely spatially auto-correlated (Global Moran’s I 0.11, z = 3.24, p < 0.01). Eastern Equine encephalitis virus was more common in the eastern half of the state (**Fig 6B**), which has abundant hardwood wetland forests; EEEV sites are also likely spatially auto-correlated (Global Moran’s I 0.22, z = 6.20, p < 1e-6). Spatial and habitat patterns for HJV isolations were similar to EEEV. The mammalian arboviruses did display some site-specific associations (**Fig 7**); however, there were no discernable ecological or spatial relationships among these sites (**Fig 7, S3 Table**) (Global Moran’s I test z-values: CVV -1.15, JCV—1.24, POTV—0.14, TVTV—0.56, all p-values > 0.2).

## Discussion

Mosquitoes and the viruses they transmit are often studied in isolation of the communities in which they coexist. This may be due to the epidemiological need to protect humans or livestock from specific threats; it may also be due to resource limitations that result in limited spatiotemporal surveillance, selective testing of only certain vector species, and screening collections for only the most medically-important arboviruses. Using a unique long-term mosquito and arbovirus surveillance data set that includes information on the abundance and infection status of all collected species, our community ecology approach extends the understanding of the generality of multi-mosquito species associations with arboviruses circulating in U.S. We find that in the northeast U.S., vector-virus associations exist along a continuum, with the avian arboviruses behaving as vector specialists (infecting a few key species in discrete habitats) and the mammalian arboviruses behaving as vector generalists (infecting numerous genera throughout a transmission season in multiple habitat types). For the avian arboviruses, which presently represent the greatest mosquito-borne disease threats to human health in the region, our results support previous findings that WNV and EEEV associate with discrete habitats which are in part strongly associated with the presence of specific species in the mosquito community [3, 36]. Additionally, our GLMM results suggest that the detection of these avian arboviral infections in predominantly mammalian biting species likely represent random exposure events. Rather than indicators of non-primary species contributions to transmission, infections in mammalian feeding species may better signify the intensity of enzootic transmission (assuming the rate at which mammalian feeding vectors encounter the avian arbovirus is proportional to the intensity of the virus’ enzootic transmission cycle). In contrast, we found that the mammalian arboviruses may rely less on specific primary vectors and more on the functional presence of mammalian feeding species in the mosquito community. Enhanced surveillance and further vector competence studies of the mammalian arboviruses are needed to better clarify the role of the mosquito community in the transmission dynamics of these viruses.

We note that there has been an increase in the number of mosquito species in CT from 32 species captured in 2001 to 43 species captured in 2018. This increase may in part be driven by recent range expansions of both native and introduced species in the U.S., such as *Ae*. *albopictus* (commonly detected beginning 2010) [37], *Aedes atlanticus* Dyar & Knab (2014), and *Culex erraticus* (Dyar & Knab) (2012). We also found that mosquito communities across CT were more dissimilar between sites than between years, supporting previous reports that local-scale processes are the primary drivers of mosquito populations [35]. The noted increase in annual mosquito species richness was not accompanied by an increase in arbovirus richness, and, unlike for mosquito communities, arbovirus communities varied as much between sites as they did years. The lack of any detected spatial and temporal arbovirus community composition patterns could be due to the existence of multiple vectors for each arbovirus whose population dynamics vary across the state. The lack of detectable patterns may also be due to other factors unique to each arbovirus, such as the heterogeneous dynamics of herd immunity in the wildlife hosts [38] and/or weather events [39] across the state.

Overall, blood feeding ecology was the strongest driver of the likelihood of a mosquito species to harbor a specific arboviral infection. For the avian arboviruses of human importance (WNV and EEEV), the majority of arboviral isolations were limited to a small subset of the avian-biting mosquito species in CT. Our analyses confirm that *Cx*. *pipiens* is the primary vector of WNV [36] and *Cs*. *melanura* is the primary vector of EEEV [40]. For WNV, certain secondary species may pose an added risk of arboviral transmission to humans besides *Cx*. *pipiens*, including *Cx*. *restuans*, *Cx*. *salinarius*, and *Cs*. *melanura*. Though all three species shared a statistical association with WNV, WNV infections in these species were most frequently detected during peak epizootic activity, and we found no evidence that these secondary vectors contributed to any significant levels of arboviral transmission during inter-enzootic periods (i.e., spring/fall) [19]. For EEEV, *Cs*. *melanura* was the only species to share a statistical association with the virus, and EEEV isolations from the remaining vector species could be used as proxies for transmission intensity rather than represent functional contributions to transmission.

The mammalian arboviruses may be much more vector generalist than their avian counterparts. The broad utilization of multiple mosquito species may explain why we found no strong spatial or habitat associations among the four analyzed mammalian arboviruses. The prevalence of mammalian arboviruses in multiple mosquito species and habitats throughout the state could reflect a number of mechanisms, none of which are mutually exclusive. White-tailed deer (*Odocoileus virginianus*) are the main blood meal hosts for the majority of these mammalian biting mosquitoes and their associated viruses [41–43], and the overabundance of deer populations across CT may explain the diffuseness of mammalian arbovirus detection patterns [20, 21]. Many of these mammalian-biting species may also be competent for multiple arboviruses, although more vector competence studies are needed to confirm this hypothesis.

Finally, our data strongly suggests that JCV associates with two functional groups of vectors. Univoltine *Aedes* spp. appear to function as overwintering reservoir hosts as well as vectors that contribute to viral persistence and early season amplification, while multivoltine *Aedes* spp. (and *An*. *punctipennis*) may act as further amplifying vectors later within a season. A similar mechanism of seasonal persistence in overwintering/amplification vectors has been proposed for Ross River virus in Australia [15]. The recent rise in the diagnosis of clinical manifestations of human disease with JCV in the upper Midwest and northeast U.S. over the last decade [1, 44] clearly indicate that more research is needed to confirm the functional importance of overwintering and amplifying vectors in the JCV transmission cycle. We additionally encourage greater scrutiny of similar overwintering/amplification predictions for WNV and other avian arboviruses [45].

The broad detection of each arbovirus across CT, especially WNV, indicates that numerous foci of transmission may exist for each arbovirus. If numerous foci of arboviral transmission exist, each could act as a source for infectious vectors and hosts and dilute the potential benefit of localized vector control [46]. Therefore, spatially limited vector control interventions may have little to no overall impact on arboviral transmission at scales relevant to public health (i.e., municipal levels such as towns or counties) [47]. The involvement of multiple mosquito species in arboviral transmission, as suggested by our analyses, will only compound the effort needed to control zoonotic arboviruses of public health importance [22]. Due to the specificity of vector-virus associations among the avian arboviruses, regional integration of vector control programs could improve the control of WNV and EEEV. However, we caution that the successes and failures of WNV/EEEV control in the northeast U.S. may have limited applicability to the control of the mammalian arboviruses.

**Data Accessibility:** Data presented in this report is available using the following URL: https://doi.org/10.5061/dryad.rjdfn2z6x

## Supporting information

S1 Table Species-specific collections and arbovirus detection summaries. (DOCX)

S2 Table Environmental fit results for the non-linear multidimensional analysis of mosquito species by collection site with land cover data for twelve classes at a 2.59 km^2^ buffer around each site. (DOCX)

S3 Table Environmental fit results for a non-linear multidimensional analysis of virus isolations by collection site with land cover data for twelve classes at a 2.59 km^2^ buffer around each site. (DOCX)

S4 Table Correlations between mosquito and arbovirus community composition metrics by sites and years. (DOCX)

S5 Table Parameter estimates from a binomial-error generalized linear mixed effects model for West Nile virus detection (1—infected, 0—uninfected) in mosquito samples collected in Connecticut from 2001–2018. Model terms included an offset for pool size, a fixed effect term for trap type, and random intercept effect terms for site, week, and year of collection as well as mosquito species identification.
(DOCX)

S6 Table Parameter estimates from a binomial-error generalized linear mixed effects model for eastern equine encephalitis virus detection (1—infected, 0—uninfected) in mosquito samples collected in Connecticut from 2001–2018. Model terms included an offset for pool size, a fixed effect term for trap type, and random intercept effect terms for site, week, and year of collection as well as mosquito species identification.
(DOCX)

S7 Table Parameter estimates from a binomial-error generalized linear mixed effects model for Jamestown Canyon virus detection (1—infected, 0—uninfected) in mosquito samples collected in Connecticut from 2001–2018. Model terms included an offset for pool size, a fixed effect term for trap type, and random intercept effect terms for site, week, and year of collection as well as mosquito species identification.
(DOCX)

S8 Table Parameter estimates from a binomial-error generalized linear mixed effects model for Cache Valley virus detection (1—infected, 0—uninfected) in mosquito samples collected in Connecticut from 2001–2018. Model terms included an offset for pool size, a fixed effect term for trap type, and random intercept effect terms for site, week, and year of collection as well as mosquito species identification.
(DOCX)

S9 Table Parameter estimates from a binomial-error generalized linear mixed effects model for Highlands J virus detection (1—infected, 0—uninfected) in mosquito samples collected in Connecticut from 2001–2018. Model terms included an offset for pool size, a fixed effect term for trap type, and random intercept effect terms for site, week, and year of collection as well as mosquito species identification.
(DOCX)

S10 Table Parameter estimates from a binomial-error generalized linear mixed effects model for Potosi virus detection (1—infected, 0—uninfected) in mosquito samples collected in Connecticut from 2001–2018. Model terms included an offset for pool size, a fixed effect term for trap type, and random intercept effect terms for site, week, and year of collection as well as mosquito species identification.
(DOCX)

S11 Table Parameter estimates from a binomial-error generalized linear mixed effects model for Trivittatus virus detection (1—infected, 0—uninfected) in mosquito samples collected in Connecticut from 2001–2018. Model terms included an offset for pool size, a fixed effect term for trap type, and random intercept effect terms for site, week, and year of collection as well as mosquito species identification.
(DOCX)

S1 Fig Nonlinear multidimensional scaling plot of the number of arbovirus isolates by mosquito species across all sampling sites and seasons: natural history parameters (in red text), species (black text and +’s), and arboviruses (blue text and x’s). Only natural history parameters variables significant at p = 0.01 are shown, and only the names of the mosquito species which odds of arbovirus detection’s 95% confidence interval were greater than unity are shown.
(TIF)

S2 Fig Color legend for Figs 4 and 5. (TIF)
