# Supplementary material for: Patterns of mosquito and arbovirus community composition and ecological indexes of arboviral risk in the northeast United States
Source: PLoS Negl Trop Dis. 2020 Feb 24;14(2):e0008066. doi: 10.1371/journal.pntd.0008066 (PMC7058363; doi:10.1371/journal.pntd.0008066)
Supplement: S1 Table — (DOCX) [file pntd.0008066.s001.docx]

| Species | Total Collection | Total Pools | N Sites | N Years | CVV | JCV | POTV | TVTV | EEE | HJV | WNV |
| --- | --- | --- | --- | --- | --- | --- | --- | --- | --- | --- | --- |
| Aedes  abserratus | 19,431 | 1,498 | 80 | 18 | 0 | 24 | 1 | 0 | 0 | 0 | 0 |
| Aedes  albopictus | 2,820 | 1,051 | 32 | 10 | 1 | 0 | 0 | 0 | 0 | 0 | 0 |
| Aedes  atlanticus | 33 | 25 | 12 | 4 | 0 | 0 | 0 | 0 | 0 | 0 | 0 |
| Aedes  atropalpus | 23 | 18 | 9 | 10 | 0 | 0 | 0 | 0 | 0 | 0 | 0 |
| Aedes  aurifer | 27,623 | 1,843 | 77 | 18 | 0 | 39 | 3 | 1 | 0 | 0 | 0 |
| Aedes  canadensis | 383,731 | 15,332 | 87 | 18 | 19 | 99 | 58 | 1 | 29 | 5 | 9 |
| Aedes  cantator | 44,345 | 4,017 | 87 | 18 | 5 | 61 | 9 | 0 | 4 | 0 | 0 |
| Aedes  cinereus | 180,015 | 13,451 | 87 | 18 | 9 | 10 | 29 | 1 | 16 | 6 | 13 |
| Aedes  communis | 401 | 52 | 21 | 13 | 0 | 2 | 0 | 0 | 0 | 0 | 0 |
| Aedes  diantaeus | 7 | 4 | 2 | 4 | 0 | 0 | 0 | 0 | 0 | 0 | 0 |
| Aedes  excrucians | 8,229 | 1,387 | 84 | 18 | 0 | 11 | 0 | 0 | 0 | 1 | 0 |
| Aedes  fitchii | 120 | 21 | 10 | 6 | 0 | 0 | 0 | 0 | 0 | 0 | 0 |
| Aedes  grossbecki | 87 | 51 | 30 | 10 | 0 | 0 | 0 | 0 | 0 | 0 | 0 |
| Aedes  hendersoni | 4 | 4 | 4 | 4 | 0 | 0 | 0 | 0 | 0 | 0 | 0 |
| Aedes  infirmatus | 28 | 18 | 9 | 1 | 0 | 0 | 0 | 0 | 0 | 0 | 0 |
| Aedes  intrudens | 2 | 2 | 2 | 1 | 0 | 0 | 0 | 0 | 0 | 0 | 0 |
| Aedes  japonicus | 27,653 | 9,966 | 87 | 18 | 1 | 0 | 0 | 0 | 0 | 0 | 10 |
| Aedes  provocans | 662 | 71 | 12 | 14 | 0 | 9 | 0 | 0 | 0 | 0 | 0 |
| Aedes  sollicitans | 21,388 | 1,413 | 46 | 18 | 4 | 5 | 2 | 0 | 1 | 0 | 1 |
| Aedes  sticticus | 59,632 | 3,085 | 87 | 18 | 0 | 18 | 14 | 2 | 1 | 0 | 2 |
| Aedes  stimulans | 18,131 | 2,519 | 86 | 18 | 0 | 20 | 3 | 0 | 0 | 0 | 2 |
| Aedes taeniorhynchus | 132,507 | 4,072 | 47 | 18 | 19 | 17 | 32 | 0 | 0 | 0 | 4 |
| Aedes  thibaulti | 104,224 | 3,986 | 86 | 15 | 0 | 3 | 5 | 1 | 0 | 0 | 0 |
| Aedes  triseriatus | 34,798 | 8,146 | 87 | 18 | 7 | 1 | 13 | 0 | 2 | 5 | 3 |
| Aedes  trivittatus | 155,178 | 9,254 | 87 | 18 | 21 | 16 | 65 | 84 | 7 | 1 | 4 |
| Aedes  vexans | 302,117 | 17,107 | 87 | 18 | 17 | 13 | 41 | 2 | 10 | 3 | 15 |
| Anopheles  barberi | 117 | 105 | 47 | 15 | 0 | 0 | 0 | 0 | 0 | 0 | 0 |
| Anopheles  crucians | 1,230 | 454 | 60 | 14 | 0 | 0 | 0 | 0 | 0 | 0 | 1 |
| Anopheles punctipennis | 51,919 | 10,414 | 87 | 18 | 49 | 37 | 47 | 1 | 5 | 1 | 5 |
| Anopheles quadrimaculatus | 11,104 | 4,006 | 87 | 18 | 5 | 2 | 4 | 0 | 2 | 1 | 1 |
| Anopheles  walkeri | 38,391 | 3,243 | 86 | 18 | 3 | 1 | 3 | 0 | 0 | 0 | 2 |
| Coquillettidia perturbans | 528,466 | 19,463 | 87 | 18 | 17 | 23 | 24 | 1 | 3 | 1 | 10 |
| Culiseta  melanura | 144,593 | 11,016 | 87 | 18 | 1 | 0 | 0 | 0 | 190 | 98 | 101 |
| Culiseta minnesotae | 486 | 162 | 30 | 17 | 0 | 0 | 0 | 0 | 0 | 0 | 0 |
| Culiseta  morsitans | 2,121 | 881 | 69 | 18 | 0 | 1 | 0 | 0 | 1 | 1 | 2 |
| Culex  erraticus | 850 | 232 | 41 | 7 | 0 | 0 | 0 | 0 | 0 | 0 | 0 |
| Culex  pipiens | 282,358 | 19,942 | 87 | 18 | 0 | 0 | 1 | 0 | 1 | 1 | 1,496 |
| Culex  restuans | 115,986 | 15,484 | 87 | 18 | 0 | 1 | 1 | 0 | 2 | 4 | 295 |
| Culex  salinarius | 227,008 | 12,929 | 87 | 18 | 2 | 3 | 7 | 0 | 9 | 3 | 150 |
| Culex  territans | 2,026 | 1,310 | 85 | 18 | 0 | 0 | 0 | 0 | 0 | 0 | 1 |
| Orthopodomyia signifera | 48 | 47 | 31 | 15 | 0 | 0 | 0 | 0 | 0 | 0 | 0 |
| Psorophora  ciliata | 17 | 9 | 4 | 3 | 0 | 0 | 0 | 0 | 0 | 0 | 0 |
| Psorophora columbiae | 82 | 45 | 14 | 10 | 0 | 0 | 0 | 0 | 0 | 0 | 0 |
| Psorophora  ferox | 118,242 | 7,404 | 87 | 18 | 10 | 1 | 8 | 1 | 2 | 2 | 5 |
| Psorophora howardii | 89 | 55 | 26 | 10 | 0 | 0 | 0 | 0 | 0 | 0 | 0 |
| Uranotaenia sapphirina | 51,941 | 7,507 | 87 | 18 | 0 | 0 | 0 | 0 | 11 | 4 | 3 |
| Virus-specific totals | | | | | | | | | | | |
| Total isolates | | | | | 190 | 417 | 370 | 95 | 297 | 137 | 2,135 |
| N years | | | | | 11 | 18 | 8 | 12 | 12 | 9 | 18 |
| N Sites | | | | | 63 | 75 | 76 | 20 | 34 | 32 | 84 |
| N species | | | | | 17 | 24 | 21 | 10 | 18 | 16 | 23 |
