# Supplementary material for: Patterns of mosquito and arbovirus community composition and ecological indexes of arboviral risk in the northeast United States
Source: PLoS Negl Trop Dis. 2020 Feb 24;14(2):e0008066. doi: 10.1371/journal.pntd.0008066 (PMC7058363; doi:10.1371/journal.pntd.0008066)
Supplement: S2 Table — (DOCX) [file pntd.0008066.s002.docx]

| NMDS Fit | | | | |
| --- | --- | --- | --- | --- |
| Distance Method | Dimensions | Stress |  |  |
| Bray – Curtis | 3 | 0.15 |  |  |
| Environmental Fit | | | | |
| Land use class | NMDS 1 | NMDS 2 | r^2^ | Pr (>r) |
| Agriculture | -0.51 | 0.86 | 0.05 | 0.11 |
| Barren | 0.89 | -0.46 | 0.23 | 1.8e-5 |
| Coniferous | -0.92 | -0.38 | 0.25 | 6.0e-6 |
| Deciduous | -0.95 | -0.32 | 0.41 | 1.0e-6 |
| Developed | 0.90 | 0.44 | 0.43 | 1.0e-6 |
| Grass | 0.56 | 0.83 | 0.22 | 2.8e-5 |
| Grass (other) | 0.04 | 0.999 | 0.001 | 0.96 |
| Utility | -0.24 | 0.97 | 0.08 | 0.04 |
| Water | 0.88 | -0.48 | 0.35 | 1.0e-6 |
| Wetland (forested) | -0.99 | -0.17 | 0.43 | 1.0e-6 |
| Wetland (non-forested) | -0.84 | 0.55 | 0.02 | 0.46 |
| Wetland (tidal) | 0.71 | -0.70 | 0.14 | 0.002 |
