# Supplementary material for: Patterns of mosquito and arbovirus community composition and ecological indexes of arboviral risk in the northeast United States
Source: PLoS Negl Trop Dis. 2020 Feb 24;14(2):e0008066. doi: 10.1371/journal.pntd.0008066 (PMC7058363; doi:10.1371/journal.pntd.0008066)
Supplement: S3 Table — (DOCX) [file pntd.0008066.s003.docx]

| NMDS Fit | | | | |
| --- | --- | --- | --- | --- |
| Distance Method | Dimensions | Stress |  |  |
| Bray – Curtis | 3 | 0.13 |  |  |
| Environmental Fit | | | | |
| Land use class | NMDS 1 | NMDS 2 | r^2^ | Pr (>r) |
| Agriculture | -0.83 | -0.55 | 0.02 | 0.50 |
| Barren | 0.30 | -0.95 | 0.03 | 0.32 |
| Coniferous | 0.01 | 0.999 | 0.03 | 0.34 |
| Deciduous | -0.998 | 0.06 | 0.06 | 0.07 |
| Developed | 1.00 | 0.002 | 0.12 | 0.005 |
| Grass | 0.83 | 0.56 | 0.05 | 0.13 |
| Grass (other) | -0.67 | 0.75 | 0.02 | 0.37 |
| Utility | 0.96 | 0.29 | 0.001 | 0.75 |
| Water | 0.01 | -0.999 | 0.04 | 0.17 |
| Wetland (forested) | -0.999 | 0.02 | 0.08 | 0.04 |
| Wetland (non-forested) | 0.13 | 0.99 | 0.01 | 0.53 |
| Wetland (tidal) | -0.32 | -0.95 | 0.002 | 0.94 |
