# Supplementary material for: Patterns of mosquito and arbovirus community composition and ecological indexes of arboviral risk in the northeast United States
Source: PLoS Negl Trop Dis. 2020 Feb 24;14(2):e0008066. doi: 10.1371/journal.pntd.0008066 (PMC7058363; doi:10.1371/journal.pntd.0008066)
Supplement: S4 Table — (DOCX) [file pntd.0008066.s004.docx]

|  | Term | Sites | Years |
| --- | --- | --- | --- |
| Community composition metrics | Richness | r = 0.09 (-0.12, 0.30)  p = 0.40 | r = -0.31 (-0.68, 0.18)  p = 0.21 |
|  | Evenness  (i.e., Shannon-Weiner index) | r = 0.07 (-0.15, 0.27)  p = 0.54 | r = 0.28 (-0.21, 0.66)  p = 0.26 |
|  | Average ecological distance | r = 0.33 (0.13, 0.51)  p = 0.002 | r = -0.28 (-0.66, 0.22)  p = 0.26 |
| Contributions to diversity | Alpha  (within unit) | r = 0.09 (-0.12, 0.30)  p = 0.40 | r = 0.31 (-0.68, 0.18)  p = 0.21 |
|  | Beta  (distinctiveness) | r = 0.10 (-0.11, 0.31)  p = 0.34 | r = -0.46 (-0.76, 0.01)  p = 0.06 |
|  | Gamma  (regional diversity) | r = 0.10 (-0.11, 0.30)  p = 0.36 | r = -0.43 (-0.75, 0.05)  p = 0.07 |
