# Supplementary material for: Patterns of mosquito and arbovirus community composition and ecological indexes of arboviral risk in the northeast United States
Source: PLoS Negl Trop Dis. 2020 Feb 24;14(2):e0008066. doi: 10.1371/journal.pntd.0008066 (PMC7058363; doi:10.1371/journal.pntd.0008066)
Supplement: S8 Table — Model terms included an offset for pool size, a fixed effect term for trap type, and random intercept effect terms for site, week, and year of collection as well as mosquito species identification. (DOCX) [file pntd.0008066.s008.docx]

| Fixed Effects | | | | |
| --- | --- | --- | --- | --- |
| Term | Estimate | Std. Error | Z value | Pr(>\|z\|) |
| Intercept | -29.8 | 11.3 | -2.63 | 0.008 |
| Light Trap | 16.8 | 11.3 | 1.49 | 0.14 |
| Random Intercept Effects | | | | |
| Group | Variance | Std. Dev. |  |  |
| Site (n = 87) | 0.38 | 0.62 |  |  |
| Species (n = 46) | 2.31 | 1.52 |  |  |
| Week (n = 18) | 0.62 | 2.48 |  |  |
| Year (n = 18) | 4.88 | 2.21 |  |  |
