# Supplementary material for: Patterns of mosquito and arbovirus community composition and ecological indexes of arboviral risk in the northeast United States
Source: PLoS Negl Trop Dis. 2020 Feb 24;14(2):e0008066. doi: 10.1371/journal.pntd.0008066 (PMC7058363; doi:10.1371/journal.pntd.0008066)
Supplement: S10 Table — Model terms included an offset for pool size, a fixed effect term for trap type, and random intercept effect terms for site, week, and year of collection as well as mosquito species identification. (DOCX) [file pntd.0008066.s010.docx]

| Fixed Effects | | | | |
| --- | --- | --- | --- | --- |
| Term | Estimate | Std. Error | Z value | Pr(>\|z\|) |
| Intercept | -14.8 | 1.61 | -9.16 | < 2e-16 |
| Light Trap | -0.16 | 0.42 | -0.38 | 0.71 |
| Random Intercept Effects | | | | |
| Group | Variance | Std. Dev. |  |  |
| Site (n = 87) | 0.28 | 0.53 |  |  |
| Species (n = 46) | 4.42 | 2.10 |  |  |
| Week (n = 18) | 6.44 | 2.54 |  |  |
| Year (n = 18) | 13.8 | 3.72 |  |  |
