# Supplementary figures and images for: Patterns of mosquito and arbovirus community composition and ecological indexes of arboviral risk in the northeast United States
Source: PLoS Negl Trop Dis. 2020 Feb 24;14(2):e0008066. doi: 10.1371/journal.pntd.0008066 (PMC7058363; doi:10.1371/journal.pntd.0008066)

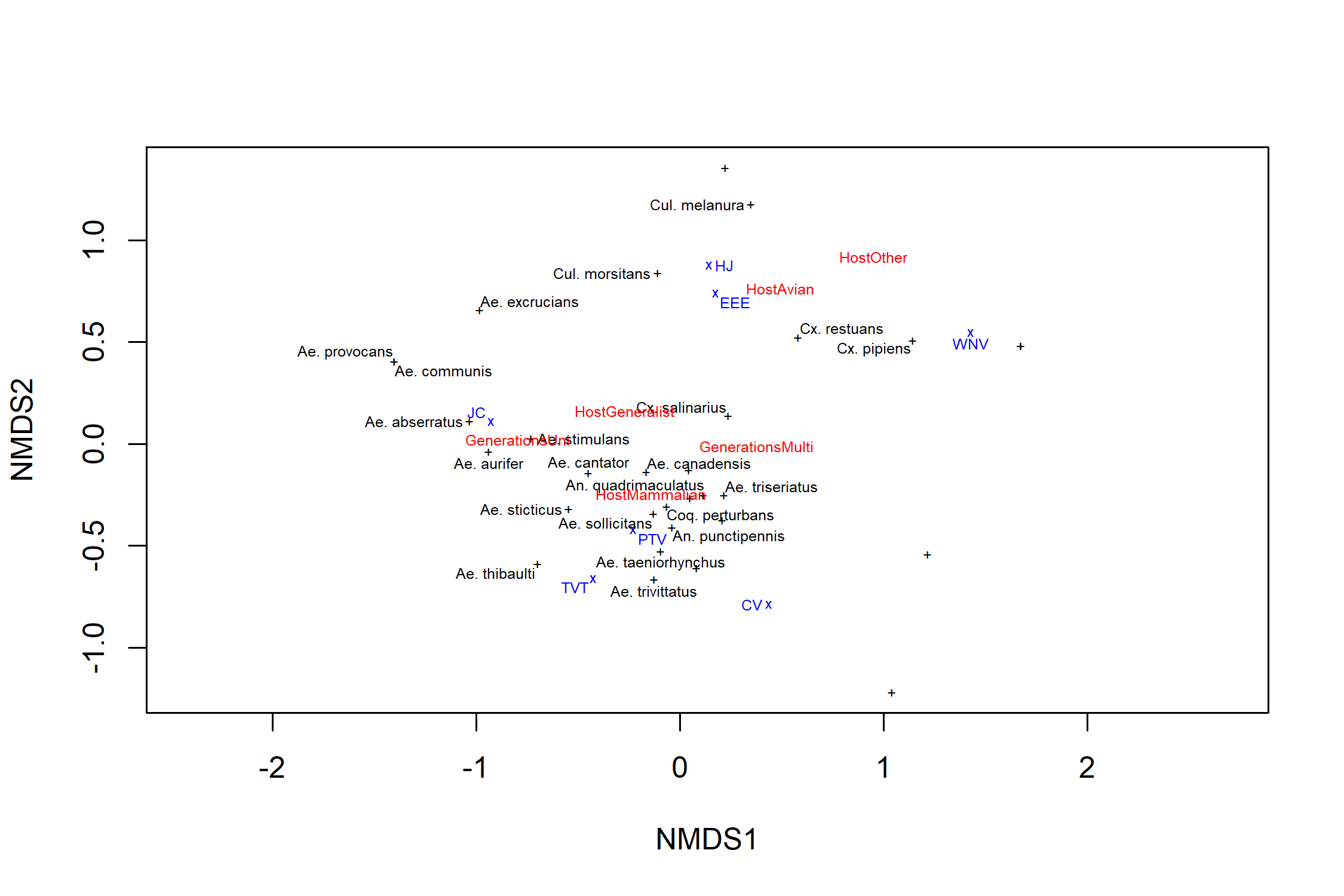

Supplement: S1 Fig — Nonlinear multidimensional scaling plot of the number of arbovirus isolates by mosquito species across all sampling sites and seasons: natural history parameters (in red text), species (black text and +’s), and arboviruses (blue text and x’s). Only natural history parameters variables significant at p = 0.01 are shown, and only the names of the mosquito species which odds of arbovirus detection’s 95% confidence interval were greater than unity are shown. (TIF) [file pntd.0008066.s012.tif]

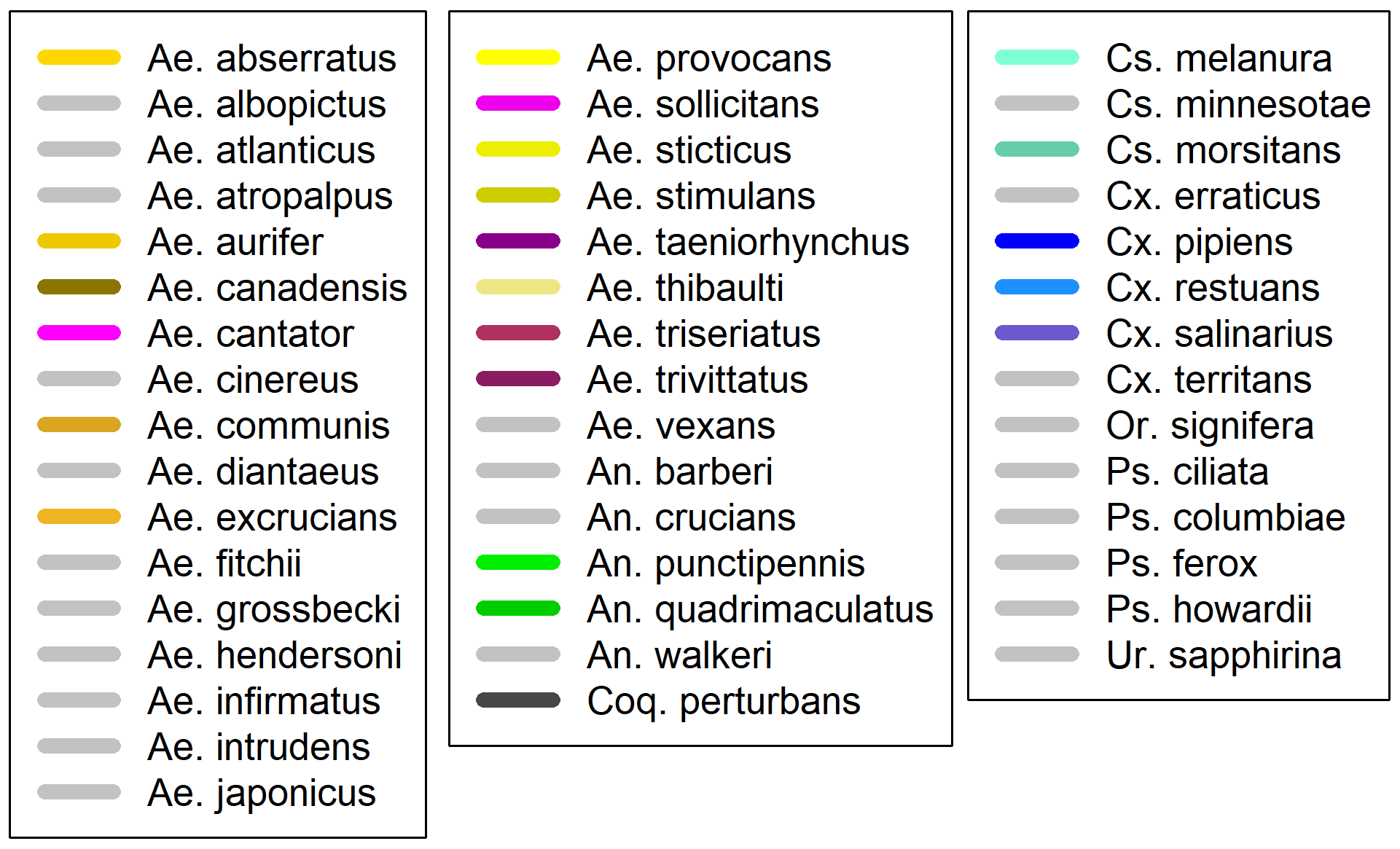

Supplement: S2 Fig — (TIF) [file pntd.0008066.s013.tif]
